# Neonatal Sepsis and Hemostasis

**DOI:** 10.3390/diagnostics12020261

**Published:** 2022-01-20

**Authors:** Dimitra Gialamprinou, Georgios Mitsiakos, Georgios N. Katsaras, Christos-Georgios Kontovazainitis, Paraskevi Karagianni, Emmanuel Roilides, Gili Kenet

**Affiliations:** 1 Second Neonatal Department and Neonatal Intensive Care Unit (NICU), “Papageorgiou” General Hospital, Medical School, Aristotle University of Thessaloniki, Nea Efkarpia, 56403 Thessaloniki, Greece; gialamprinou@gmail.com (D.G.); gkatsaras84@gmail.com (G.N.K.); chgkontovazainitis@gmail.com (C.-G.K.); 2 First Neonatal Department and Neonatal Intensive Care Unit (NICU), “Hippokration” General Hospital, Medical School, Aristotle University of Thessaloniki, 54642 Thessaloniki, Greece; karagpar@gmail.com; 3 Third Department of Paediatrics, Hippokration University Hospital, Medical School, Aristotle University of Thessaloniki, 54642 Thessaloniki, Greece; roilides@gmail.com; 4 National Hemophilia Center, Thrombosis Unit and Amalia Biron Research Institute of Thrombosis and Hemostasis, Sheba Medical Center, Tel-Hashomer 52621, Israel; Gili.kenet@sheba.health.gov.il; 5 Sackler School of Medicine, Tel Aviv University, Tel Aviv 6997801, Israel

**Keywords:** neonatal sepsis, inflammation, hemostasis, platelets, viscoelastic tests, flow cytometry

## Abstract

Neonatal sepsis is considered critical for a significant increase in neonatal morbidity and mortality among hospitalized neonates. Neonatal sepsis, in most cases, coexists with coagulopathy, which can prove to be life-threatening. Complex molecular and cellular systems are involved in the cross-talk between inflammation and hemostasis during sepsis. Disturbances in the regulating systems of the vascular endothelium, and platelet–endothelial and platelet–neutrophil interactions play a pivotal role in both inflammation and coagulation. This complex process is poorly understood in neonates. In addition to the developmental maturation of hemostasis and the immune response in neonatal sepsis, a cellular model of hemostasis during sepsis should be taken into account. This review focused on the molecular and cellular mechanisms underlying inflammation and hemostasis during neonatal sepsis, taking the developmental immune response and developmental hemostasis into account in order to provide future diagnostic approaches to be applied in everyday clinical settings. Regarding the diagnostic modalities, we briefly provide the limitations of the currently used conventional coagulation assays, focusing on viscoelastic tests and platelet flow cytometry.

## 1. Introduction

Neonatal sepsis is the third most common cause of newborn mortality [1]. To date, the reported fatalities from neonatal sepsis have reached a rate of 2% in full-term infants and 20% in preterm infants, and it was further complicated by hemodynamic, inflammatory and clinical instability associated with systemic inflammatory response syndrome (SIRS) and meningitis in 30% of cases [2,3,4,5].

Sepsis is frequently related to coagulation disorders and presents with varied severity. In sepsis, both hemostasis and the immune system are activated, sharing common pathways that respond to cellular–endothelium interactions, eventually resulting in microvascular thrombosis. This complex process of thrombosis formation is poorly understood in adults and not at all in neonates [6,7]. Neonatal sepsis can present as mildly prolonged coagulation times and mild thrombocytopenia. However, it is often accompanied by significant and sometimes life-threatening coagulopathy, as well as by disseminated intravascular coagulopathy and thromboembolic events that impair organ function and fulminant bleeding that require prompt and appropriate intervention [8,9,10].

This review focuses on the molecular and cellular mechanisms underlying inflammation and hemostasis during neonatal sepsis, presents the main developmental differences between neonatal and adult hemostasis and platelet function, briefly reviews the currently available hemostatic tests in neonates and their limitations, and provides future diagnostic approaches to be applied in everyday clinical settings. Particularly, we considered the viscoelastic tests and platelet flow cytometry.

## 2. Developmental Hemostasis in Neonates

Age-related variations in coagulation components have been well established since the introduction of developmental hemostasis by Andrew et al., and were further validated by Monagle et al. in healthy full-term neonates and children up to 16 years old [11]. Compared with those of older individuals, the coagulation components and the properties of the endothelium in neonates are strongly affected by age [12,13]. Coagulation factors are produced by the fetus starting at 11 weeks’ gestation, and elevated levels are observed as gestation progresses and in postnatal life. Subsequently, preterm neonates exhibit lower coagulation factor levels compared with older individuals and adults. At birth, the vitamin K-dependent coagulation factors are almost at 30–50% of the adult levels for extremely preterm and full-term neonates, respectively, and reach adult values by approximately 6 months of age [14,15]. In contrast, coagulation factors V, VIII (FV, FVIII), and XIII, and von Willebrand factor (vWF) reach almost normal adult levels at birth [16].

The levels of natural anticoagulants, namely antithrombin (antithrombin-III, AT), heparin cofactor II, and proteins C and S, are significantly reduced in both preterm and full-term neonates, reaching almost 50% of adult levels at birth, except for α-macroglobulin, which is markedly increased. These coagulation inhibitors progressively increase near to adult levels by 3 and 6 months, respectively, for full-term and preterm neonates [14,15]. Furthermore, the activity of the plasmin/plasminogen system decreases, along with reduced fibrinolysis and faster thrombin generation capacity to ensure the neonatal hemostasis balance, was detected in preterm neonates in particular [17].

Furthermore, the antithrombotic and fibrinolytic properties of the endothelium are related to age; consequently, the neonatal endothelium is likely to differ from that of adults. In particular, endothelial cells express selectins in an age-dependent pattern similar to developmental hemostasis. Specifically, E-selectin and P-selectin reach adult levels by 32 and 11 weeks’ gestation [18]. Neonatal endothelial cells exhibit a low capacity to reverse oxidative agents, while lower levels of molecules with adhesion properties have been recorded at distinct gestational and postnatal ages [19]. Researchers have shed some light on the glycocalyx in neonates with necrotizing enterocolitis, but the biological structure of the endothelium and glycocalyx components in neonates is still under investigation [20].

Fetal platelets originate in megakaryocytes located in the fetal liver and, in the first trimester of pregnancy, already account for almost 150,000/μL. At 22 to 24 weeks’ gestation, they reach a stable value of around 250,000/μL until delivery in full-term gestation [21]. Additionally, the hyporeactivity of platelets has been reported in neonates during the first 10 days of life, while platelet counts are similar in neonates and adults. A significant impairment in activation and aggregation capability was found in neonatal platelets from cord blood after stimulation with adenosine diphosphate (ADP), epinephrine, collagen, thrombin, and thromboxane analogs in vitro compared with adult platelets [22]. This deficit is dominant among preterm neonates in response to platelet agonists [20]. Neonatal platelets present a reduced number of a2-adrenergic receptors, reduced expression of the thrombin receptors (protease activated receptors, PARs) PAR-1 and PAR-4, and a downregulation of signaling from the thromboxane receptor. Age-related hypersensitivity in the effect of prostaglandin E1 (PGE1) has been observed [23]. In addition, neonatal platelets’ hyporesponsiveness can be attributed to fewer dense granules and functional defects in the alpha granules, which decrease the degranulation and the fibrinogen-binding capacity [24]. Interestingly, counteractive factors, such as the mean platelet volume (MCV), higher hematocrit at birth, and higher concentrations of vWf and its ultra-large multimers, enhance the interaction between platelets and vessels and ultimately keep the neonatal hemostatic status in balance.

However, healthy neonates preserve coagulation homeostasis by maintaining balanced procoagulant protein levels in the plasma, thrombin generation, and fibrinolysis capacity, along with platelet hyporeactivity during the first days of life [25,26]. In contrast to their healthy counterparts, the fine line between bleeding and clotting is apparently disturbed in sick neonates, particularly during episodes of sepsis.

## 3. Neonatal Sepsis and Hemostasis

Inflammation and coagulation are mutually regulated [27]. Cytokines and chemokines, especially interleukin (IL) 6 and tumor necrosis factor alpha (TNF-α), which are expressed early at the onset and throughout the duration of sepsis, trigger the coagulation process through a cascade that leads to the activation of coagulation factors and anticoagulant proteins. In particular, the proinflammatory cytokines activate coagulation through upregulation of tissue factor (TF) expression and TF-mediated thrombin generation, and also through downregulation of the protein C system and enhanced fibrinolysis inhibition [6]. Coagulation factors interact with PARs, thus contributing to the inflammatory process. Elements of hemostasis, mainly thrombin, the TF-VIIa complex, and factor Xa, bind to PARs and activate the inflammatory response via intracellular endothelial cell signaling [28]. This is an excellent model for supporting the claim that inflammation promotes coagulation, after which coagulation intensifies the inflammatory process. Although this has been sufficiently elucidated in adults, it is still being investigated in neonates.

Animal studies have shown that T cells during fetal and neonatal life present a rather “toleragenic” (a TH2-skewed response) and anti-inflammatory phenotype, which is consistent with the developmental maturation of hemopoietic stem cells, and that neonates are highly prone to infection, in accordance with gestational age and postnatal health status [29]. Neonatal animal models presented with a weakened inflammatory response, including downregulation of the TNF-a related genes and a defective production of pro-inflammatory cytokines IL-23, IL-6, and IL-10, along with lower absolute concentrations of plasma cytokines and chemokines namely IL-1a/b, IL-12, granulocyte-macrophage colony-stimulating factor and sargramostim (GM-CSF), macrophage inflammatory protein (MIP)-1b, and IFN g [26].

During toxinemia, TF is expressed in the macrophages and monocytes, and initiates thrombin generation, thus promoting clot formation via extracellular vesicles. The TF contained in microparticles circulating in the plasma is transferred to cells that do not produce it or produce it in part, such as platelets, thereby enhancing the coagulation cascade during sepsis. This activation process arises from procoagulant cytokines, mainly IL-1 and TNF-a [30]. Neonates with an early predominance of inflammatory cytokines during sepsis have an increased risk of developing DIC [31]. This finding is consistent with the elevated levels of IL-6 in the serum and the high frequency of DIC seen with disseminated viral infection [2].

In order to counteract the spread of clotting, antithrombin, alongside its anticoagulation properties, provides downregulation of the cytokine receptors by linking to inflammatory cells [32]. Antithrombin interacts with endothelial cells, monocytes, neutrophils, and lymphocytes, and enhances prostacyclin release. The latter inhibits the interaction between endothelial cells and inflammatory cells, and also reduces the production of various cytokines and chemokines by endothelial cells [33]. In addition, activation of tissue factor induces an increase in the levels of the thrombin/antithrombin III (ATIII) complex (TAT), the plasminogen activator inhibitor (PAI), and the plasmin-α2–antiplasmin complex (PAP). Elevated levels of TAT and PAP in septic VLBW infants at 26–32 weeks’ gestation have been recorded, but no clear trend towards either thrombosis or hemorrhage has been shown [34]. In a prospective cohort study of full-term neonates with sepsis, severe infection was associated with activation of the contact system and consumption of anticoagulant proteins, in parallel with increased levels of the proteins of the complement system. Moreover, protein S was inactivated and anticoagulant proteins, including the TAT complex, increased, while fibrinolysis was inhibited, establishing a hypercoagulable state which resolved after antibiotic therapy among survivors [35]. Similarly, in a prospective case–control study, a significant simultaneous reduction in the TAT complex and protein C with elevated levels of inactive protein S was observed in full-term neonates with confirmed sepsis, while protein C levels were most markedly reduced in those who fatally developed DIC [36]. It has also been proven that neonates in the early stages of sepsis are prone to a prothrombotic state due to the consumption of coagulation inhibitors and activation of the coagulation cascade through cytokine release. This hypercoagulable state could mostly be resolved after administration of appropriate therapy. As there is ample evidence to show that activated protein C induces a reduction in TNF-α, IL-1β, IL-6, and IL-8 by blocking monocytes/macrophages, protein C has been suggested as potential therapeutic agent in neonatal sepsis [37]. In children with sepsis, fibrinolysis is profoundly inhibited, which is mostly attributed to an increase in plasma activity of the fibrinolysis inhibitor plasminogen activator inhibitor-1 (PAI-1) during sepsis, leading to severe sepsis and septic shock. Plasminogen activator inhibitor-1 has also been proposed as a promising treatment in pediatric sepsis, but relevant studies in neonates are still lacking [38].

Sepsis-induced endothelial dysfunction expressed as the disruption of antithrombotic properties results in the accumulation of fibrinogen [39]. During sepsis, disturbance in the endothelial glycocalyx structure modulates endothelium–neutrophil–platelet interactions, leading to thrombus formation and also to exacerbated fibrin formation and circulatory disorders. Glycocalyx impairment, along with inflammation during sepsis, leads to capillary leakage and vascular damage, which enhances inflammation and hypercoagulation. These aberrations result in increased vascular permeability, altered blood flow, impaired oxygen delivery, and ultimately to organ dysfunction [40]. Disturbances in the endothelial glycocalyx function induces disorders in tissue factor activation; thus disturbing the production of the tissue-type plasminogen activator and the plasminogen activator inhibitor-1. Moreover, the expression of glycosaminoglycans (such as heparan sulfates) of the injured glycocalyx is also diminished. Recently, reduced levels of endothelial glycocalyx components have been highlighted for reducing the hemostatic response of the endothelium. Furthermore, in adults, syndecan-1 levels are associated with the severity of sepsis and the development of DIC [41]. The attenuation of the anticoagulant properties of the glycosaminoglycans will directly impair the anticoagulant effect of the endothelium. Consequently, the fine line between thrombosis and bleeding becomes apparent [42]. Compared with adults, a decrease in TNF-a production from endothelial cells after inflammatory stimulation was shown in neonatal mouse models with *Pneumocystis carinii* infection of the respiratory tract. The diminished TNF-a production failed to enhance the expression of adhesion molecules in the surface of endothelial cells and finally attenuated the T cells’ migration capacity and the host’s defense response to infection [43]. This point underlines the importance of molecules deriving from endothelial cells and the glycocalyx during neonatal sepsis. In this context, TNFα has been proposed as a potential immunomodulator in neonatal sepsis [43]. Certainly, strong evidence regarding the contribution of the glycocalyx and endothelial cells to managing neonatal sepsis is still lacking, but this is a fairly promising research field.

## 4. Neonatal Platelets in Sepsis

Platelets are major players in sepsis-induced coagulopathy. During systemic inflammation, P-selectin is expressed on the platelet surface, facilitating the platelets’ adhesion to leukocytes and platelet aggregation, in parallel with tissue factor expression on monocytes [44]. Recently, the enhanced expression of GPIIb/IIIa receptors on activated platelet surfaces has been recognized in association with infection by *Staphylococcus aureus* and *Escherichia coli*, thereby demonstrating direct platelet activation in response to bacterial invasion and simultaneously introducing thromboinflammation and immunothrombosis [45,46,47]. The etiopathology and management of immunothrombosis in infancy and early childhood still lack sufficient evidence, as little research has been conducted in the pediatric population [48].

Thrombocytopenia noted in septic patients is mainly attributed to platelet consumption during clot propagation and thrombus formation through the activated endothelium [49]. In neonates, thrombocytopenia driven by LPS in Gram-negative sepsis is thought to be related to diminished expression of platelet Toll-like receptor 4 (TLR4) and is linked to elevated mortality rates [50]. Platelet activation was associated with high expression of platelet CD40L following endothelium inflammation, and higher platelet aggregation was observed after LPS stimulation (mostly in Gram-negative sepsis) [51]. Higher CD40L expression levels in platelets from cord blood samples were observed in premature neonates with histologically proven chorioamnionitis [52].

Moreover, research on neonatal thrombopoiesis during sepsis strongly suggested that neonates respond to sepsis by upregulating thrombopoietin (Tpo) production; although the degree of upregulation is modest, neonates present with a hypercoagulant profile at the onset of infection. In septic neonates, elevated levels of circulating megakaryocyte progenitors (CMPs) have also been observed. Simultaneous measurements of serum TPO levels and reticulated platelets (RP%) are helpful for discriminating hyperdestructive from hypoplastic thrombocytopenia in septic neonates [53,54].

As thrombocytopenia is linked to increased morbidity and mortality in ICU admissions, the delineation of platelet functionality may potentially alter the threshold levels of platelet transfusions [55]. Septic preterm neonates, when compared with healthy individuals, present a lower platelet adhesion capacity, which is mostly attributed to deficiencies in the intrinsic platelet properties rather than to an impairment in the concentrations or function of vWf [50]. These studies demonstrated that platelet activation and degranulation may follow thrombocytopenia, and that this phenomenon should be further investigated by means of accurate qualitative modalities for mapping distinct platelet phenotypes in patients with sepsis.

The missing part of this complex interplay between inflammation and coagulation is quantification of this model of cell-based coagulation triggered by an agent, such as sepsis, by means of a validated and practical tool for use in everyday clinical settings.

## 5. Coagulation Monitoring in Neonates

Platelet count and plasma-based measurements of clot formation have been at the center of long-established modalities of hemostasis testing. However, platelet function and activity have not been included in this conventional approach. Moreover, plasma-based techniques present reduced sensitivity in cases of defects in fibrinolysis, as well as in states of augmented clot figuration. Additionally, traditional testing of hemostasis ignores the significance of endothelium and platelet function and the interaction among the endothelium, platelets, inflammation, and coagulation. Additionally, there is a need for a more detailed view of the process of clot formation and fibrinolysis over the course of time. There has definitely been notable progress in these directions; recent studies have conducted assessments of thrombin formation in the plasma and have also focused on clarifying the steps of hemostasis in whole blood. Especially for critically ill infants, the diagnostic approach and optimal management entail an adequate comprehension of clot formation and the establishment of hemostasis, together with identification of the tests’ powers and inadequacies.

The most widely used modalities to assess in vivo clot formation focus on the synthesis of fibrin from fibrinogen and use different methodologies with a wide range of sensitivity to estimate the time of clot formation. Prothrombin time (PT), activated partial thromboplastin time (aPTT), and thrombin time (TT) represent the most common tests. In these tests, the parameter “time to clot formation” is the common element of the so-called “functional assays” and is based on normal adult plasma. These tests appear to be simple and can be easily standardized; however, they can be impacted by both in vitro and in vivo parameters that do not actually act upon in vivo clot formation. Neonates, particularly premature ones, stand as an example of the above, as less plasma is contained in a blood sample which is over-anticoagulated. Neonates, in the vast majority of cases, present with steadily prolonged clotting times, as PT and aPTT reflect the lower activity levels of the coagulation proteins in the neonatal population and not an increased bleeding risk [56].

Moreover, the results from studies on the developmental maturation of neonatal hemostasis question the validity of applying age-related reference ranges for monitoring the neonatal hemostatic system [11]. However, the discrepancies between neonatal coagulation reference ranges and standard coagulation assays extrapolated from adult values make the evaluation of the disease and the choice of therapeutic management a considerable challenge.

## 6. Viscoelastic Tests

A considerable number of important assays that assess hemostasis have emerged in clinical practice. Viscoelastic tests examine the development of clots in whole blood in a dynamic way. This approach mainly includes thromboelastography (TEG), rapid thromboelastography (r-TEG), and rotational thromboelastometry (ROTEM). In these modalities, only a small amount of blood is required, which is put into a cup together with a pin that acts as a probe. Viscoelastic testing analyzes the process of thrombosis, from the initial steps of clot formation to resolution of the thrombus, including the interim stages of clot development as well as clot stability. These clot elements are assessed with the use of measurements recorded in a short course of time.

Thromboelastography and ROTEM are widely used in managing surgical patients with trauma-associated coagulopathy. They are applied to detect hyperfibrinolysis and guide the management of bleeding with targeted blood products in trauma coagulopathy [57]. Fibrinolysis is also disrupted primarily in severely septic patients with developed DIC and is strongly correlated with increased morbidity and mortality in severe sepsis and septic shock [58,59]. The essence of thromboelastic testing in patients presenting with hemorrhage has been recently described by a Cochrane review that encompassed 17 trials, two of which included children. These studies showed that transfusion guided by TEG/ROTEM could potentially lead to fewer transfusions with additional favorable mortality results. Further research in the pediatric population is also being conducted with the aim of examining the use of viscoelastic testing in the optimal management of individuals with non-inherited disorders of coagulation or those mandating anticoagulation [60].

The utility of TEG/ROTEM tests for a more individualized transfusion policy to guide treatment in patients with hemorrhage is well established. TEG/ROTEM testing has been carried out extensively in cases of in adult sepsis and, although they are not yet understood well in neonates, they are promising tools for future research and clinical use in this patient population as well [61].

To the best of our knowledge, there are only few relevant studies assessing hemostatic changes in septic neonates. Hypocoagulability profiles have been detected by Grant and Hadley by means of TEG in 27 neonates who underwent surgery with established or early sepsis, and the derived coagulation hyporesponsiveness was consistent with the pathophysiology of coagulation consumption during severe sepsis and DIC [62]. Sokou et al.’s study used rotational thromboelastometry to describe coagulopathy disorders in severely septic neonates [63]. The pathology appeared as an abnormal pattern of prolonged clot initiation or clot formation, and narrow amplitudes indicative of defective clot strength were detected in TEG as well. An intense hypocoagulation profile was correlated with more severe stages in DIC or bleeding diathesis. Conversely, a ROTEM pattern of suspected sepsis or of mild or early stage infection appeared as hypercoagulability, suggestive of the pathophysiology of coagulation-compensating mechanisms for restricting the spread of infection, although cohort studies could be more informative regarding the underlying time-dependent mechanisms during sepsis. Interestingly, the parameters of ROTEM or TEG, such as the maximum clot firmness (MCF) or lysis index 60 (LI60), are affected by fibrinogen and platelet counts, and they can adequately reflect the interaction between fibrinolysis and platelets [64]. As all blood components and their interactions in fibrin generation and dissolution can be accurately and quickly assessed, thromboelastometry variables might serve for estimating the prognosis of neonatal sepsis [65].

The current literature provides case–control studies on the initial stage of neonatal sepsis, but the findings of a cohort study on neonatal sepsis that used ROTEM/TEG could provide a useful tool for early identification of the appropriate interventions that target blood components in cases of severe sepsis or DIC [66]. The significance of studying the evolution of hemostatic processes during sepsis was described in a recent prospective study of 55 cases of pediatric sepsis and DIC. In this cohort study, the patients fulfilled the criteria of septic shock. At the early point of diagnosis, ROTEM revealed various coagulation profiles, ranging from normal coagulation and hypercoagulation to a strongly hypocoagulable state. Nevertheless, patients with overt DIC and non-survivors showed prolonged clotting time (CT) and clotting firmness time (CFT), and reductions in the α-angle (α), MCF, and thrombodynamic potential index (TPI); these findings were compatible with hypocoagulation. The hypocoagulation profile was predictive of mortality. Interestingly, the hypercoagulable tendency was shown by ROTEM, but conventional testing failed to provide clear evidence of hypercoagulability. The alterations in the hemostatic profile were clear and determined the time-point for intervention in the event of impending complications, namely guided transfusion in the case of bleeding [67]. The main limitation of the validated use of these modalities is the inability to assess platelet function. They also lack accuracy in cases of low platelet counts and severe thrombocytopenia.

## 7. Platelet Function Assessment

Platelet function, in association with platelet surface receptor expression under stimulation, has been investigated by flow cytometry. In adult studies, flow cytometry prove to be a highly sensitive modality for revealing smooth differences in platelet function among diverse patient subgroups [68,69]. This method, although considered relatively reproducible, mainly depends upon staff expertise and the choice of agonists and antibodies. It has been used in the neonatal population and showed clear differences among individuals. Furthermore, in comparison with adults, the findings suggested the lower expression of platelet surface receptors and the hyporeactivity of neonatal platelets [70]. There is only a limited number of surveys focusing on platelet function in preterm neonates using flow cytometry, and these present high heterogeneity regarding the study design. However, although the available data are restricted, they clearly suggest the presence of differences in platelet function, mainly associated with gestational age and very low birth weight. It is thought that this observation correlates with the “developmental progression” of hemopoiesis, which has inherent differences in the platelet progenitors [71,72].

Hemorrhage in preterm neonates is often observed in parallel with thrombocytopenia, without any apparent causality between platelet count and bleeding risk [73]. Thrombocytopenia does not stand alone as a risk factor for bleeding events, and thus there is a need to develop laboratory tests to provide a better correlation with bleeding risk other than platelet count alone, especially tests that focus mainly on platelet function. Flow cytometry is a highly accurate method for the analysis of platelet function, and—mainly in comparison with aggregometry—it requires a small volume of blood for the sample, a feature which is of special significance for neonates with an extremely low birth weight [74,75]. Unlike aggregometry, flow cytometry is a method with high accuracy for the evaluation of single cells separately while also being informative for a large number of platelets derived from small amounts of blood [76]. Platelet functionality is assessed by the expression of platelet surface receptors, granule release, and the change in platelet shape in comparison with aggregometry, in which aggregation capability is imperfectly assessed and for which a relatively large amount of blood is required. Additionally, flow cytometry is not affected by the presence of thrombocytopenia. With regard to the platelet function analyzer (PFA-100/PFA-200), the in vitro bleeding time provides an unclear association between platelet function and bleeding risk. Although the application of flow cytometry requires specialized laboratory facilities and a well-trained staff, a recent study on neonates born at 27–41 weeks of gestation described an adaptation of flow cytometry for bedside application [77]. Those authors identified differences in platelet function among separate neonatal groups, and provided a potential use of the above method in the evaluation of neonates requiring platelet transfusion. Of special note, they developed degranulation scores as well as fibrinogen-binding scores that clearly differentiated between healthy neonates and septic neonates, and claimed that platelet function consisted of risk factors for the development of intraventricular hemorrhage and necrotizing enterocolitis.

## 8. Viscoelastic Tests and Platelet Function

In an effort to overcome the gaps in laboratory investigations of hemostasis disorders in neonates, the first attempt to evaluate clot formation simultaneously with the platelets’ functional activity was performed with the use of thrombodynamics and flow cytometry, as described by Koltsova et al. [78]. Their study was performed on healthy full-term and late preterm newborns, and platelet hyporesponsiveness was combined with hypercoagulation profiles modified by gestational age. An evaluation of clot formation, together with platelet function among adults and children transfused with platelets after a cardiopulmonary bypass, has recently been reported [79]. The discrepancy between MCF in the extrinsic (EXTEM) and fibrinogen (FIBTEM) pathway reaction times of ROTEM tests was recently used as an index for platelets’ contribution to clot strength [80]. The difference in clot elasticity between these variables may be a more accurate index of the platelets’ contribution to clot formation. In a recent study, the main research question was the correlation of clot elasticity with platelet count and platelet function, as evaluated by platelet function tests. Specifically, platelet aggregation was evaluated after activation by adenosine diphosphate and thrombin receptor activating peptide using multiple electrode aggregometry (the ADP test and TRAP test of the Multiplate Analyzer) [80].

## 9. Conclusions

Apparently, neonatal sepsis coexists with coagulopathy, which ranges from mild coagulation disorders and hypercoagulability with little or no clinical impact to severe hypocoagulability as detected in septic shock and bleeding in DIC. ROTEM has been established as a sensitive modality for the detection of hypo- or hypercoagulation in bleeding patients, as well as in septic patients. Flow cytometry is a method that is reproducible enough to provide clear differences in platelet function among individuals.

To date, there have been no studies on the identification of hemostasis disorders by means of viscoelastic tests in combination with platelet flow cytometry in sick neonates. It could be possible that the simultaneous interpretation of viscoelastic variables and flow cytometry outcomes may be correlated to bleeding risk and may therefore guide the administration of plasma and platelet transfusion. A prospective cohort study representative of gestational age and disease status is needed, wherein bleeding scores would be recorded in parallel with flow cytometry and ROTEM data. This design could make the resulting data compatible with use in everyday clinical settings by validating the combination of ROTEM measurements with platelet function and platelet counts in order to revise and enhance the current transfusion thresholds.

## References

[1] GBD 2015 Mortality and Causes of Death Collaborators (2016). Global, Regional, and National Life Expectancy, All-Cause Mortality, and Cause-Specific Mortality for 249 Causes of Death, 1980–2015: A Systematic Analysis for the Global Burden of Disease Study 2015. Lancet.

[2] Wynn J.L., Wong H.R. (2010). Pathophysiology and Treatment of Septic Shock in Neonates. Clin. Perinatol..

[3] Wynn J.L. (2016). Defining Neonatal Sepsis. Curr. Opin. Pediatr..

[4] Kim F., Polin R.A., Hooven T.A. (2020). Neonatal Sepsis. BMJ.

[5] Shane A.L., Sánchez P.J., Stoll B.J. (2017). Neonatal Sepsis. Lancet.

[6] Van der Poll T., Levi M. (2012). Crosstalk between Inflammation and Coagulation: The Lessons of Sepsis. Curr. Vasc. Pharmacol..

[7] Raymond S.L., Stortz J.A., Mira J.C., Larson S.D., Wynn J.L., Moldawer L.L. (2017). Immunological Defects in Neonatal Sepsis and Potential Therapeutic Approaches. Front. Pediatr..

[8] Iba T., Levy J.H. (2020). Sepsis-Induced Coagulopathy and Disseminated Intravascular Coagulation. Anesthesiology.

[9] Gando S., Levi M., Toh C.-H. (2016). Disseminated Intravascular Coagulation. Nat. Rev. Dis. Prim..

[10] Levi M. (2008). The Coagulant Response in Sepsis. Clin. Chest Med..

[11] Monagle P., Barnes C., Ignjatovic V., Furmedge J., Newall F., Chan A., De Rosa L., Hamilton S., Ragg P., Robinson S. (2006). Developmental Haemostasis. Impact for Clinical Haemostasis Laboratories. Thromb. Haemost..

[12] Wisgrill L., Muck M., Wessely I., Berger A., Spittler A., Förster-Waldl E., Sadeghi K. (2018). Endothelial Cells of Extremely Premature Infants Display Impaired Immune Response after Proinflammatory Stimulation. Pediatr. Res..

[13] Pietrasanta C., Pugni L., Ronchi A., Bottino I., Ghirardi B., Sanchez-Schmitz G., Borriello F., Mosca F., Levy O. (2019). Vascular Endothelium in Neonatal Sepsis: Basic Mechanisms and Translational Opportunities. Front. Pediatr..

[14] Andrew M., Paes B., Milner R., Johnston M., Mitchell L., Tollefsen D.M., Powers P. (1987). Development of the Human Coagulation System in the Full-Term Infant. Blood.

[15] Andrew M., Paes B., Milner R., Johnston M., Mitchell L., Tollefsen D.M., Castle V., Powers P. (1988). Development of the Human Coagulation System in the Healthy Premature Infant. Blood.

[16] Andrew M., Vegh P., Johnston M., Bowker J., Ofosu F., Mitchell L. (1992). Maturation of the Hemostatic System during Childhood. Blood.

[17] Toulon P., Berruyer M., Brionne-François M., Grand F., Lasne D., Telion C., Arcizet J., Giacomello R., De Pooter N. (2016). Age Dependency for Coagulation Parameters in Paediatric Populations. Results of a Multicentre Study Aimed at Defining the Age-Specific Reference Ranges. Thromb. Haemost..

[18] Auvinen K., Jalkanen S., Salmi M. (2014). Expression and Function of Endothelial Selectins during Human Development. Immunology.

[19] Huet O., Dupic L., Harrois A., Duranteau J. (2011). Oxidative Stress and Endothelial Dysfunction during Sepsis. Front. Biosci. (Landmark Ed.).

[20] Karatepe H.O., Kilincaslan H., Berber M., Ozen A., Saricoban H.E., Ustek D., Kemik A.S., Adas M., Bakar F. (2014). The Effect of Vascular Endothelial Growth Factor Overexpression in Experimental Necrotizing Enterocolitis. Pediatr. Surg. Int..

[21] Forestier F., Daffos F., Catherine N., Renard M., Andreux J.P. (1991). Developmental Hematopoiesis in Normal Human Fetal Blood. Blood.

[22] Sitaru A.G., Holzhauer S., Speer C.P., Singer D., Obergfell A., Walter U., Grossmann R. (2005). Neonatal Platelets from Cord Blood and Peripheral Blood. Platelets.

[23] Palma-Barqueros V., Torregrosa J.M., Caparrós-Pérez E., Mota-Pérez N., Bohdan N., Llanos M.D.C., Begonja A.J., Sola-Visner M., Vicente V., Teruel-Montoya R. (2020). Developmental Differences in Platelet Inhibition Response to Prostaglandin E1. Neonatology.

[24] Schlagenhauf A., Schweintzger S., Birner-Grünberger R., Leschnik B., Muntean W. (2010). Comparative Evaluation of PAR1, GPIb-IX-V, and Integrin AIIbβ3 Levels in Cord and Adult Platelets. Hamostaseologie.

[25] Williams M.D., Chalmers E.A., Gibson B.E.S. (2002). The Investigation and Management of Neonatal Haemostasis and Thrombosis. Br. J. Haematol..

[26] Sola-Visner M. (2012). Platelets in the Neonatal Period: Developmental Differences in Platelet Production, Function, and Hemostasis and the Potential Impact of Therapies. Hematol. Am. Soc. Hematol. Educ. Progr..

[27] Levi M., van der Poll T., Büller H.R. (2004). Bidirectional Relation between Inflammation and Coagulation. Circulation.

[28] Levi M., van der Poll T. (2017). Coagulation and Sepsis. Thromb. Res..

[29] Harbeson D., Francis F., Bao W., Amenyogbe N.A., Kollmann T.R. (2018). Energy Demands of Early Life Drive a Disease Tolerant Phenotype and Dictate Outcome in Neonatal Bacterial Sepsis. Front. Immunol..

[30] Iba T., Ogura H. (2018). Role of Extracellular Vesicles in the Development of Sepsis-Induced Coagulopathy. J. Intensive Care.

[31] Ng P.C., Li K., Leung T.F., Wong R.P.O., Li G., Chui K.M., Wong E., Cheng F.W.T., Fok T.F. (2006). Early Prediction of Sepsis-Induced Disseminated Intravascular Coagulation with Interleukin-10, Interleukin-6, and RANTES in Preterm Infants. Clin. Chem..

[32] Levy J.H., Sniecinski R.M., Welsby I.J., Levi M. (2016). Antithrombin: Anti-Inflammatory Properties and Clinical Applications. Thromb. Haemost..

[33] Gierer P., Laue F., Hoffmann J.N., Rotter R., Mittlmeier T., Gradl G., Vollmar B. (2013). Antithrombin Reduces Inflammation and Microcirculatory Perfusion Failure in Closed Soft-Tissue Injury and Endotoxemia. Crit. Care Med..

[34] Aronis S., Platokouki H., Photopoulos S., Adamtziki E., Xanthou M. (1998). Indications of Coagulation and/or Fibrinolytic System Activation in Healthy and Sick Very-Low-Birth-Weight Neonates. Biol. Neonate.

[35] Roman J., Velasco F., Fernandez F., Fernandez M., Villalba R., Rubio V., Vicente A., Torres A. (1993). Coagulation, Fibrinolytic and Kallikrein Systems in Neonates with Uncomplicated Sepsis and Septic Shock. Haemostasis.

[36] El Beshlawy A., Alaraby I., Abou Hussein H., Abou-Elew H.H., Mohamed Abdel Kader M.S.E. (2010). Study of Protein C, Protein S, and Antithrombin III in Newborns with Sepsis. Pediatr. Crit. Care Med. J. Soc. Crit. Care Med. World Fed. Pediatr. Intensive Crit. Care Soc..

[37] Lauterbach R., Wilk B., Bocheńska A., Hurkała J., Radziszewska R. (2016). Nonactivated Protein C in the Treatment of Neonatal Sepsis: A Retrospective Analysis of Outcome. Pediatr. Infect. Dis. J..

[38] Green J., Doughty L., Kaplan S.S., Sasser H., Carcillo J.A. (2002). The Tissue Factor and Plasminogen Activator Inhibitor Type-1 Response in Pediatric Sepsis-Induced Multiple Organ Failure. Thromb. Haemost..

[39] Schött U., Solomon C., Fries D., Bentzer P. (2016). The Endothelial Glycocalyx and Its Disruption, Protection and Regeneration: A Narrative Review. Scand. J. Trauma. Resusc. Emerg. Med..

[40] Iba T., Levy J.H. (2019). Derangement of the Endothelial Glycocalyx in Sepsis. J. Thromb. Haemost..

[41] Ikeda M., Matsumoto H., Ogura H., Hirose T., Shimizu K., Yamamoto K., Maruyama I., Shimazu T. (2018). Circulating Syndecan-1 Predicts the Development of Disseminated Intravascular Coagulation in Patients with Sepsis. J. Crit. Care.

[42] Fourrier F. (2012). Severe Sepsis, Coagulation, and Fibrinolysis: Dead End or One Way?. Crit. Care Med..

[43] Qureshi M.H., Cook-Mills J., Doherty D.E., Garvy B.A. (2003). TNF-Alpha-Dependent ICAM-1- and VCAM-1-Mediated Inflammatory Responses Are Delayed in Neonatal Mice Infected with Pneumocystis Carinii. J. Immunol..

[44] Tunjungputri R.N., van de Heijden W., Urbanus R.T., de Groot P.G., van der Ven A., de Mast Q. (2017). Higher Platelet Reactivity and Platelet-Monocyte Complex Formation in Gram-Positive Sepsis Compared to Gram-Negative Sepsis. Platelets.

[45] Arman M., Krauel K., Tilley D.O., Weber C., Cox D., Greinacher A., Kerrigan S.W., Watson S.P. (2014). Amplification of Bacteria-Induced Platelet Activation Is Triggered by FcγRIIA, Integrin AIIbβ3, and Platelet Factor 4. Blood.

[46] Watson C.N., Kerrigan S.W., Cox D., Henderson I.R., Watson S.P., Arman M. (2016). Human Platelet Activation by Escherichia Coli: Roles for FcγRIIA and Integrin AIIbβ3. Platelets.

[47] Martinod K., Deppermann C. (2021). Immunothrombosis and Thromboinflammation in Host Defense and Disease. Platelets.

[48] Franchi T., Eaton S., De Coppi P., Giuliani S. (2019). The Emerging Role of Immunothrombosis in Paediatric Conditions. Pediatr. Res..

[49] Iba T., Levy J.H. (2018). Inflammation and Thrombosis: Roles of Neutrophils, Platelets and Endothelial Cells and Their Interactions in Thrombus Formation during Sepsis. J. Thromb. Haemost..

[50] Finkelstein Y., Shenkman B., Sirota L., Vishne T.H., Dardik R., Varon D., Linder N. (2002). Whole Blood Platelet Deposition on Extracellular Matrix under Flow Conditions in Preterm Neonatal Sepsis. Eur. J. Pediatr..

[51] Henn V., Slupsky J.R., Gräfe M., Anagnostopoulos I., Förster R., Müller-Berghaus G., Kroczek R.A. (1998). CD40 Ligand on Activated Platelets Triggers an Inflammatory Reaction of Endothelial Cells. Nature.

[52] Sitaru A.-G., Speer C.P., Holzhauer S., Obergfell A., Walter U., Grossmann R. (2005). Chorioamnionitis Is Associated with Increased CD40L Expression on Cord Blood Platelets. Thromb. Haemost..

[53] Eissa D.S., El-Farrash R.A. (2013). New Insights into Thrombopoiesis in Neonatal Sepsis. Platelets.

[54] Brown R.E., Rimsza L.M., Pastos K., Young L., Saxonhouse M.A., Bailey M., Lawrence R.M., Sola-Visner M.C. (2008). Effects of Sepsis on Neonatal Thrombopoiesis. Pediatr. Res..

[55] Thorup C.V., Christensen S., Hvas A.-M. (2020). Immature Platelets As a Predictor of Disease Severity and Mortality in Sepsis and Septic Shock: A Systematic Review. Semin. Thromb. Hemost..

[56] Pal S., Curley A., Stanworth S.J. (2015). Interpretation of Clotting Tests in the Neonate. Arch. Dis. Child. Fetal Neonatal Ed..

[57] Da Luz L.T., Nascimento B., Shankarakutty A.K., Rizoli S., Adhikari N.K. (2014). Effect of Thromboelastography (TEG^®^) and Rotational Thromboelastometry (ROTEM^®^) on Diagnosis of Coagulopathy, Transfusion Guidance and Mortality in Trauma: Descriptive Systematic Review. Crit. Care.

[58] Daudel F., Kessler U., Folly H., Lienert J.S., Takala J., Jakob S.M. (2009). Thromboelastometry for the Assessment of Coagulation Abnormalities in Early and Established Adult Sepsis: A Prospective Cohort Study. Crit. Care.

[59] Kim S.M., Kim S.-I., Yu G., Kim J.-S., Hong S.I., Chae B., Shin Y.S., Kim Y.J., Jang S., Kim W.Y. (2020). Role of Thromboelastography as an Early Predictor of Disseminated Intravascular Coagulation in Patients with Septic Shock. J. Clin. Med..

[60] Wikkelsø A., Wetterslev J., Møller A.M., Afshari A. (2016). Thromboelastography (TEG) or Thromboelastometry (ROTEM) to Monitor Haemostatic Treatment versus Usual Care in Adults or Children with Bleeding. Cochrane Database Syst. Rev..

[61] Katsaras G.Ν., Sokou R., Tsantes A.G., Piovani D., Bonovas S., Konstantinidi A., Ioakeimidis G., Parastatidou S., Gialamprinou D., Makrogianni A. (2021). The Use of Thromboelastography (TEG) and Rotational Thromboelastometry (ROTEM) in Neonates: A Systematic Review. Eur. J. Pediatr..

[62] Grant H.W., Hadley G.P. (1997). Prediction of Neonatal Sepsis by Thromboelastography. Pediatr. Surg. Int..

[63] Sokou R., Giallouros G., Konstantinidi A., Pantavou K., Nikolopoulos G., Bonovas S., Lytras T., Kyriakou E., Lambadaridis I., Gounaris A. (2018). Thromboelastometry for Diagnosis of Neonatal Sepsis-Associated Coagulopathy: An Observational Study. Eur. J. Pediatr..

[64] Solomon C., Ranucci M., Hochleitner G., Schöchl H., Schlimp C.J. (2015). Assessing the Methodology for Calculating Platelet Contribution to Clot Strength (Platelet Component) in Thromboelastometry and Thrombelastography. Anesth. Analg..

[65] Lampridou M., Sokou R., Tsantes A.G., Theodoraki M., Konstantinidi A., Ioakeimidis G., Bonovas S., Politou M., Valsami S., Iliodromiti Z. (2020). ROTEM Diagnostic Capacity for Measuring Fibrinolysis in Neonatal Sepsis. Thromb. Res..

[66] Boscolo A., Spiezia L., Campello E., Consolaro E.M., Ori C., Simioni P. (2020). The Prognostic Role of ThromboDynamic Index in Patients with Severe Sepsis. Intern. Emerg. Med..

[67] Tuan T.A., Ha N.T.T., Xoay T.D., My T.T.K., Nghiem L.T., Dien T.M. (2021). Hypocoagulable Tendency on Thromboelastometry Associated With Severity and Anticoagulation Timing in Pediatric Septic Shock: A Prospective Observational Study. Front. Pediatr..

[68] Rubak P., Nissen P.H., Kristensen S.D., Hvas A.-M. (2016). Investigation of Platelet Function and Platelet Disorders Using Flow Cytometry. Platelets.

[69] Garner S.F., Furnell A., Kahan B.C., Jones C.I., Attwood A., Harrison P., Kelly A.M., Goodall A.H., Cardigan R., Ouwehand W.H. (2017). Platelet Responses to Agonists in a Cohort of Highly Characterised Platelet Donors Are Consistent over Time. Vox Sang..

[70] Strauss T., Sidlik-Muskatel R., Kenet G. (2011). Developmental Hemostasis: Primary Hemostasis and Evaluation of Platelet Function in Neonates. Semin. Fetal Neonatal Med..

[71] Margraf A., Nussbaum C., Rohwedder I., Klapproth S., Kurz A.R.M., Florian A., Wiebking V., Pircher J., Pruenster M., Immler R. (2017). Maturation of Platelet Function During Murine Fetal Development In Vivo. Arterioscler. Thromb. Vasc. Biol..

[72] Margraf A., Nussbaum C., Sperandio M. (2019). Ontogeny of Platelet Function. Blood Adv..

[73] Grevsen A.K., Hviid C.V.B., Hansen A.K., Hvas A.-M. (2020). The Role of Platelets in Premature Neonates with Intraventricular Hemorrhage: A Systematic Review and Meta-Analysis. Semin. Thromb. Hemost..

[74] Huskens D., Sang Y., Konings J., van der Vorm L., de Laat B., Kelchtermans H., Roest M. (2018). Standardization and Reference Ranges for Whole Blood Platelet Function Measurements Using a Flow Cytometric Platelet Activation Test. PLoS ONE.

[75] Jurk K., Shiravand Y. (2021). Platelet Phenotyping and Function Testing in Thrombocytopenia. J. Clin. Med..

[76] Huskens D., Li L., Florin L., de Kesel P., de Laat B., Roest M., Devreese K.M.J. (2020). Flow Cytometric Analysis of Platelet Function to Improve the Recognition of Thrombocytopathy. Thromb. Res..

[77] Waller A.K., Lantos L., Sammut A., Salgin B., McKinney H., Foster H.R., Kriek N., Gibbins J.M., Stanworth S.J., Garner S.F. (2019). Flow Cytometry for Near-Patient Testing in Premature Neonates Reveals Variation in Platelet Function: A Novel Approach to Guide Platelet Transfusion. Pediatr. Res..

[78] Koltsova E.M., Balashova E.N., Ignatova A.A., Poletaev A.V., Polokhov D.M., Kuprash A.D., Ionov O.V., Kirtbaya A.R., Lenyushkina A.A., Timofeeva L.A. (2019). Impaired Platelet Activity and Hypercoagulation in Healthy Term and Moderately Preterm Newborns during the Early Neonatal Period. Pediatr. Res..

[79] Barker E.E., Saini A., Gazit A.Z., Shea S.M., Baltagi S., Gage B.F., Spinella P.C. (2019). TEG Platelet Mapping and Impedance Aggregometry to Predict Platelet Transfusion During Cardiopulmonary Bypass in Pediatric Patients. Front. Pediatr..

[80] Ranucci M., Di Dedda U., Baryshnikova E. (2020). Platelet Contribution to Clot Strength in Thromboelastometry: Count, Function, or Both?. Platelets.

